# Bibliography

**Published:** 1892-01

**Authors:** 


### Bibliography.

THE PHYSICIAN'S VISITING-LIST. Forty-first year of its publication. P. Blakiston, Son & Co., Philadelphia, 1892.

This well-known visiting-list is again presented, calculated for 1892.

Besides the metric, dose, and posological tables, it gives a list of new remedies, poisons and antidotes, disinfectants, etc.

Its general arrangement makes it one of the most convenient lists published.

Pearson's Dental Appointment-Book. R. I. Pearson & Co., Kansas City, Missouri.

This is a very convenient pocket engagement-book, with room for eight appointments for the day, with spaces for hour and operation. The date of the month has necessarily to be inserted, a serious objection common to all of this class of special publications.

A Calendar for 1892, Designed in Colors. Lee & Shepard, Boston, Mass.

This beautiful calendar is composed of heavy gilt-edged cards fastened by a silvered chain. It is one of the most artistic of the many productions of this character which come to us at this period of the year, and indicates a growing taste in this direction worthy of special commendation.

An Abstract of the Symptoms, with the Latest Dietetic and Medicinal Treatment of Various Diseased Conditions. The Food Products, Digestion and Assimilation. Reed & Carnrick, New York, 1891.

This is intended to place in the hands of the active practitioner, in a condensed form, the more recent views in relation to digestion and assimilation. And although published in the way of business, contains much valuable material of special interest.

